# Biological degradation of aflatoxin M_1_ by *Bacillus pumilus* E‐1‐1‐1

**DOI:** 10.1002/mbo3.663

**Published:** 2018-08-31

**Authors:** Xinxi Gu, Jilu Sun, Yuqi Cui, Xianghong Wang, Yaxin Sang

**Affiliations:** ^1^ College of Food Science and Technology Hebei Agricultural University Baoding China

**Keywords:** aflatoxin M_1_, *Bacillus pumilus*, biodegradation, detoxification, food safety

## Abstract

Aflatoxin M_1_ (AFM_1_) is a potent mycotoxin which causes serious health concerns in developing countries, where it is mainly found in milk, meat, and other foods. Biological detoxification is a promising method for eliminating AFM_1_. The aim of this work was to search for AFM_1_‐degrading bacterial strains from animal waste, soil, and activated sludge. High‐performance liquid chromatography and Fourier‐transform infrared spectroscopy were used to analyze the AFM_1_ degradation products. A strain designated E‐1‐1‐1 was obtained from African elephants feces, with the degradation ratio of AFM_1_ reaching 89.55% in 12 hr. Based on morphology, physiological and biochemical tests, and 16S rRNA gene sequence analysis, strain E‐1‐1‐1 was identified as *Bacillus pumilus*. The culture supernatant of *B. pumilus* E‐1‐1‐1 degraded AFM_1_ effectively, whereas the cells and cell extracts of *B. pumilus* E‐1‐1‐1 were far less effective. Carbon and nitrogen sources had highly significant effects on the degradation of AFM_1_ by *B. pumilus* E‐1‐1‐1. The AFM_1_‐degrading strain, *B. pumilus* E1‐1‐1, could have great potential in industrial applications.

## 1 INTRODUCTION

Over the past several decades, interest in a group of toxic secondary metabolites produced by *Aspergillus flavus*,* Aspergillus nomius,* and *Aspergillus parasiticus* (Peltonen, Elnezami, Haskard, Ahokas, & Salminen, 2001) and known as aflatoxins has increased due to health, economic and food safety concerns (Kumar, Mahato, Kamle, Mohanta, & Kang, 2017). Aflatoxins include B1, B2, G1, G2, M1, M2, GM1, and GM2 (Kimiko & Hiromitsu, 2013). When aflatoxin B_1_ is ingested by some food‐producing animals, its metabolites, which include aflatoxin M_1_ (AFM_1_), would be transferred to milk, eggs, meat, and so on (Charoenpornsook & Kavisarasai, 2006). Food being contaminated with AFM_1_ is observed with increasing regularity. Thus, some countries have mandated a maximum residue limit from 25 to 50 ng/kg. To date, 60 countries have established regulatory limits for AFM_1_.

Strategies for eliminating aflatoxins in contaminated commodities include physical, chemical, and biological approaches. Various chemical and physical methods have been developed for removal of AFM_1_. However, these methods have many weaknesses, such as the high cost, the loss of nutrition and so on. Biological degradation of aflatoxins is an efficient, specific, and environmentally friendly approach to reduce or eliminate the aflatoxins in foods and feeds (Wu et al., 2009). Many researchers are entering the field of biodetoxification, and furthermore, the process of biological detoxification is considered a promising method for eliminating AFM_1_ (Jebali et al., 2015; Kabak & Var, 2004).

In this study, an AFM_1_‐degrading strain, *Bacillus pumilus* E1‐1‐1, was isolated from feces samples of African elephants. As far as we know, this is the first study to report an AFM_1_‐degrading strain of *B. pumilus*.

## 2 MATERIALS AND METHODS

### 2.1 Chemicals and media

AFM_1_ was obtained from Fermentek Corporation (Israel). The DNA purification kit and LA Taq DNA polymerase were purchased from TaKaRa (Otsu, Japan). The DNA Mini kit was purchased from Omega Bio‐Tek (Norcross, GA). Coumarin medium (CM) included (per L): beef extract 3 g, NaCl 10 g, glucose 6 g, and peptone 5 g. Second screening medium contained beef extract (0.3%, w/v), NaCl (1%, w/v), glucose (0.6%, w/v), and peptone (0.5%, w/v), and the cultures were incubated with AFM_1_. All other chemicals used were of analytical reagent grade and were obtained from Sigma (St. Louis, MO).

### 2.2 Isolation of AFM_1_‐degrading strains

Forty‐two samples were collected from animal waste, soil, and activated sludge in Baoding (Hebei, China). Five grams of each sample were individually cultured in 50 ml sterilized CM at 37°C for 72 hr for the enrichment and isolation of AFM_1_‐degrading strains (Guan et al., 2008). Single colonies were chosen for further research.

The strains obtained from preliminary screening were transferred to second screening medium and incubated at 37°C for 24 hr. The fermentation liquid (0.9 ml) and AFM_1_ (0.1 ml, 400 ng/ml) were added to sterile tubes. After incubation at 37°C for 72 hr, residual AFM_1_ was measured according to the standard method. The strains that had the capacity of degrading AFM_1_ were target strains. All strains were stored at −80°C before use. A strain which showed the greatest ability to degrade AFM1 was selected for further analysis.

### 2.3 Identification of the isolates

Biochemical analysis, physiological tests, and 16S rRNA gene sequence analysis were carried out to identify the isolate. The Biolog automated bacterial identification system was utilized to test the general physiological and biochemical characteristics of the strain. Genomic DNA of the strain was extracted using the method as described previously (Hesham, 2014). A universal primer set consisting of 27F and 1492R was used to amplify the 16S rRNA gene (Lane, 1991; Topp et al., 2000). The nucleotide sequence was determined by direct sequencing and compared with available 16S rRNA gene sequences in the GenBank database using the BLAST program (National Library of Medicine, Bethesda, MD). A phylogenetic tree was reconstructed using MEGA 5.0 software (Kumar, Nei, Dudley, & Tamura, 2008).

### 2.4 Degradation of AFM_1_ by strain E‐1‐1‐1 in liquid cultures

The isolated strain was cultured in Luria‐Broth medium (LB) containing AFM_1_ (400 ng/ml) at 37°C for 14 hr. The AFM_1_ degradation experiment was carried out by inoculating 900 μl of the culture in 24 ml LB containing 100 μl AFM_1_ (400 ng/ml), final concentration of 40 ng/ml, and subsequently incubating at 37°C for 0, 2, 4, 6, 8, 10, and 12 hr. The AFM_1_ residue in the medium was determined by high‐performance liquid chromatography (HPLC). Sterile LB containing AFM_1_ was used as a control.

### 2.5 AFM_1_ degradation by cell‐free supernatant, cells and cell extracts

Strain E‐1‐1‐1 was incubated at 37°C for 24 hr in LB medium. The induced cultures were centrifuged at 12,000 *g* for 20 min at 4°C to collect cells. The cells were washed three times with phosphate buffer (pH 7.0). The cells and heat‐killed cells were incubated with AFM_1_ (100 μl, 400 ng/ml) at 37°C for 12 hr, respectively. Residual AFM_1_ was measured by HPLC.

For analysis of cell extracts, strain E‐1‐1‐1 was incubated at 37°C for 24 hr in the LB medium. The cultures were centrifuged at 4°C, 12,000 *g* for 20 min to collect cells. The cells were washed three times in phosphate buffer (pH 7.0), and then disrupted by ultrasonic vibration for 10 min. The intracellular extracts (900 μl) were incubated with AFM_1_ (100 μl, 400 ng/ml) at 37°C for 12 hr, and residual AFM_1_ was measured by HPLC.

For analysis of cell‐free supernatants, strain E‐1‐1‐1 was incubated at 37°C for 24 hr in the LB medium. The cultures were centrifuged at 4°C, 12,000 *g* for 20 min to remove cells and undissolved materials. The cell‐free supernatants (900 μl) were incubated with AFM_1_ (100 μl, 400 ng/ml) at 37°C for 12 hr, and residual AFM_1_ was measured by HPLC. Sterile LB with AFM_1_ (100 μl, 400 ng/ml) was used as a control.

### 2.6 Effect of carbon and nitrogen sources on degradation of AFM_1_

After cultivation of strain E‐1‐1‐1 in LBC medium or LBN medium at 37°C for 14 hr, the cell supernatant was collected by centrifugation (6000 *g*, 10 min, 4°C) and tested for AFM_1_ degradation ratio in different nitrogen (N) sources and different carbon (C) sources. The LBC medium was supplemented with yeast extract (5 g/L), tryptone (10 g/L), NaCl (5 g/L), and different carbon sources: glucose, fructose, sucrose, maltose, lactose, soluble starch, and trehalose at a final concentration of 6 g/L. The LBN medium was supplemented with glucose (6 g/L), NaCl (5 g/L), and different nitrogen sources in place of yeast extract and tryptone: peptone, yeast extract, beef extract, tryptone, NH_4_NO_3_, casein and Mix N (tryptone:NH_4_NO_3_, 1:1) at a final concentration of 10 g/L.

### 2.7 Isolation of AFM_1_ degradation products

Before analysis by HPLC, the samples were extracted: 900 μl of sample was incubated with 100 μl AFM_1_ in sterile tubes at 37°C for 12 hr; in the control, sterile water with AFM_1_ (100 μl, 400 ng/ml) was used. Five milliliters of methanol‐water (70:30, v/v) was added to the sample tubes and was extracted three times with chloroform (5 ml). The lower layer fluid was collected and dried by rotary evaporation at 35°C. The dried sample was dissolved in 1 ml acetonitrile solvent.

### 2.8 HPLC analysis of AFM_1_ determination

Before analysis by HPLC, the samples were extracted: 900 μl of sample was incubated with 100 μl AFM_1_ in sterile tubes at 37°C for 12 hr; in the control, sterile water with AFM_1_ (100 μl, 400 ng/ml) was used. Five milliliters of methanol‐water (70:30, v/v) was added to the sample tubes and was extracted three times with chloroform (5 ml). The lower layer fluid was collected and dried by rotary evaporation at 35°C. The dried sample was dissolved in 1 ml acetonitrile solvent for HPLC analysis.

Liquid chromatography was performed on a Waters HPLC system (Waters) equipped with a Waters 2475 Series auto sampler and a Waters 2475 fluorescence detector. The excitation and emission wavelengths were 360 and 410 nm, respectively. The stationary phase was an Agilent silica gel C18 (250 × 4.6 mm, 10 μm) column (Agilent Associates). The mobile phase was isocratic, acetonitrile:water (25:75, v/v) with a flow rate of 1 ml/min. The calibration curve was determined using a series of acetonitrile dilutions containing different amounts of AFM_1_.The dried samples were dissolved in 1 ml acetonitrile solvent, and 10 μl of the sample was injected into the HPLC system.

The detoxification ratio of AFM_1_ was calculated using the following formula: *Y* = (1 − *X*_1_/*X*_2_) × 100%, where *X*_1_ is the AFM_1_ content in the treated group, *X*_2_ is the AFM_1_ content in the negative control, and *Y* is the detoxification ratio

### 2.9 Statistical analysis

All assays in this study were carried out in triplicate. Data analysis was performed using SAS software (SAS Institute Inc., Gary, NC).

## 3 RESULTS

### 3.1 Screening of AFM_1_‐degrading strains

In this research, 68 strains showing AFM_1_ degradation were isolated from the 42 samples. These strains could grow well in CM (Table 1). Forty‐seven strains were isolated from animal feces, which had many intestinal floras that could decompose feed; eight strains were isolated from sludge; and 13 strains were isolated from soil. The strains obtained were used to analyze the detoxification ratio of AFM_1_. Ten isolates showed >60% detoxification ratio of AFM_1_ (Table 2). The strain E‐1‐1‐1 showed the strongest ability to degrade AFM_1_, with an AFM_1_ degradation percentage of 89.55%. Therefore, strain E‐1‐1‐1 was chosen for further study.

**Table 1:** Results of the preliminary screening of aflatoxin M_1_ degradation strains

Number strain	Strain source	Number strain	Strain source
2	African elephants feces	1	Roe deer feces
2	Argali feces	3	Camel feces
1	Sika deer feces	1	Arabian oryx feces
3	Guanaco feces	1	Partridge feces
2	Hippopotamus feces	2	Brown bear feces
2	African aoudad sheep feces	2	Presbytis Francois feces
1	Kinkajou feces	3	Dama feces
1	Zebra feces	2	Wapiti feces
1	Pony feces	1	Panda feces
1	Addax feces	2	Bharal feces
1	Giraffe feces	1	Baboon feces
8	Sludge	6	Deep soil feces
7	Surface soil	1	Black bear feces
2	Chimpanzee feces	3	Elk feces
3	Assamese Macaque feces	1	Cougar feces

**Table 2:** Results of the second screened aflatoxin degradation strains

Strain number	Strain source	Aflatoxin M_1_ degradation ratio (%)
E‐1‐1‐1	African elephants	89.55 ± 0.04
S‐2‐1‐1	Argali	75.63 ± 0.03
D‐2‐1‐2	Dama	71.49 ± 0.02
SO‐3‐4‐1	Deep soil	70.86 ± 0.03
W‐5‐1‐1	Sludge	69.71 ± 0.02
E‐3‐1‐1	African elephants	68.01 ± 0.04
L‐2‐1‐1	Cougar	67.69 ± 0.01
W‐1‐5‐2‐2	Sludge	66.09 ± 0.03
W‐1‐3‐2‐1	Sludge	65.09 ± 0.02
SO‐2‐3‐1	Surface soil	64.86 ± 0.02

### 3.2 Identification of strain E‐1‐1‐1

**Figure 1:**
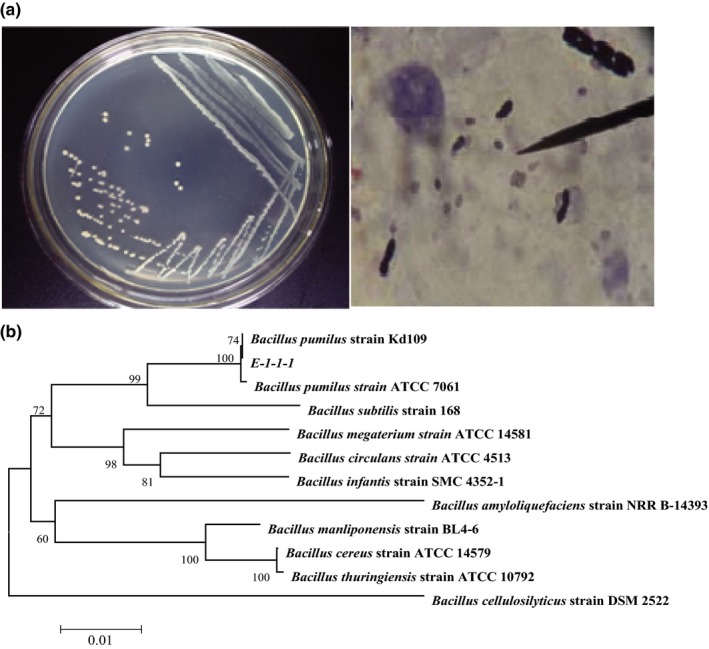
Phylogenetic relationship between strain E‐1‐1‐1 and other strains based on 16S rRNA gene sequence analysis (a) Colonial and morphology of strain E‐1‐1‐1. (b) Phylogenetic tree of strain E‐1‐1‐1 based on 16S rRNA sequence.

Colonies of strain E‐1‐1‐1 were round, small and ivory‐colored, the fringe was trim, and the surface was wet. Cells of strain E‐1‐1‐1 were short pole and obtuse round under the microscope (Figure 1a). Gram staining was positive. According to physiological and biochemical tests, strain E‐1‐1‐1 was most similar to *B. pumilus* (Table 3). The online BLAST search of the 16S rRNA gene sequence of strain E‐1‐1‐1 showed that strain E‐1‐1‐1 exhibited 99% similarity to *B. pumilus*. A phylogenetic tree was reconstructed with the 16S rRNA gene sequence of strain E‐1‐1‐1 (Figure 1b), and strain E‐1‐1‐1 was included in the cluster of the *B. pumilus* group. Based on the results of morphology, physiological, and biochemical tests, and 16S rRNA gene sequence analysis, strain E‐1‐1‐1 was finally identified as *B. pumilus* E‐1‐1‐1 (GenBank accession number: HQ423168.1).

**Table 3:** Bacterial identification

Substrate	Result	Substrate	Result	Substrate	Result	Substrate	Result
Negative control	−	Dextrin	/	d‐Maltose	/	d‐Trehalose	/
d‐Cellobiose	/	Gentiobiose	+	Sucrose	+	Turanose	+
Stachyose	−	Positive control	+	pH6	+	pH5	+
d‐Raffinose	+	a‐d‐Lactose	−	d‐Melibiose	−	β‐Methyl‐d‐Glucoside	/
d‐Salicin	/	*N*‐Acetyl‐d‐Glucosa	+	*N*‐Acety‐β‐d‐Mannosamine	/	*N*‐Acetyl‐d‐Galactosamine	−
*N*‐Acetyl Neuralminicacid	−	*N*‐Acetyl‐d‐Glucosa mine	+	4% NaCl	+	8% NaCl	+
a‐d‐Glucose	+	1% NaCl	/	d‐Fructose	+	d‐Galactose	/
3‐Methyl‐d‐Glucose	−	d‐Fucose	/	l‐Fucose	/	l‐Rhamnose	+
Inosine	−	1% Sodium lactate	+	Fusidic acid	−	d‐Serine	−
d‐Sorbitol	−	d‐Mannitol	+	d‐Arabitol	−	Myo‐inositol	−
Glycerol	+	d‐Glucose‐6‐PO4	/	d‐Fructose‐6‐PO4	/	d‐Aspartic acid	+
d‐Serine	−	Troleandomycin	−	Rifamycin SV	−	Minocycline	−
Gelatin	/	Glycyl‐l‐Proline	−	l‐Alanine	/	l‐Arginine	+
l‐Aspartic acid	+	l‐Glutamic Acid	−	l‐Histidine	−	l‐Pyroglutamic	−
l‐Serine	+	Lincomycin	−	Guanidine HCl	+	Niaproof 4	−
Pectin	+	d‐Galacturonic acid	/	l‐Galactonic acid lactone	−	d‐Gluconic acid	/
d‐Glucuronic acid	+	Glucuronamide	/	Mucic acid	−	Quinic acid	+
d‐Saccharic acid	−	Vancomycin	−	Tetrazolium violet	/	Tetrazolium blue	−
p‐Hydroxy‐phenylacetic acid	−	Methyl pyruvate	/	d‐Lactic acid methyl ester	−	l‐Lactic acid	/
Citric acid	+	α‐Keto‐glutaric acid	−	d‐Malic acid	−	l‐Malic acid	+
Bromosuccinic acid	+	Nalidixic acid	/	Lithium chloride	+	Potassium tellurite	+
Tween 40	/	γ‐Amino‐Butyric acid	/	a‐Hydroxy‐butyric acid	−	β‐Hydroxy‐D, l‐Butyric acid	−
a‐Keto‐butyric acid	−	Acetoacetic acid	/	Propionic acid	−	Acetic acid	−
Formic acid	−	Aztreonam	+	Sodium butyrate	+	Sodium bromate	−

*Note* +: positive reaction; −: negative reaction;/: infirmness reaction.

### 3.3 AFM_1_ degradation by B. pumilus

**Figure 2:**
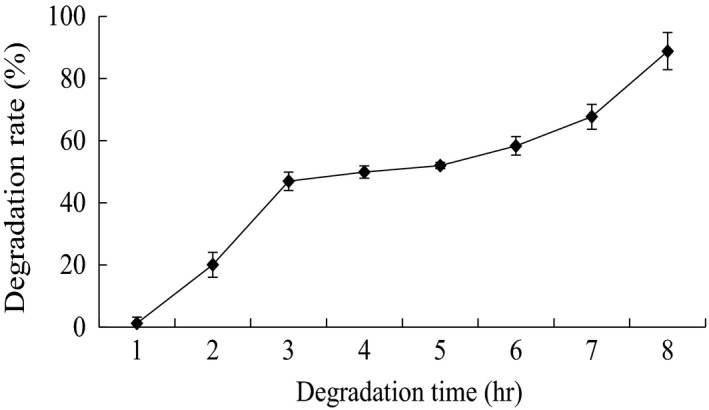
Change curve of aflatoxin M_1_ degradation ratio by strain E‐1‐1‐1 culture supernatant with time

AFM_1_ degradation by strain E‐1‐1‐1 after different time intervals was studied (Figure 2). The result showed the AFM_1_ degradation ratio increased with incubation time. The degradation ratio of AFM_1_ could reach 50% in the first 2 hr, while the maximum value of degradation ratio (89.55%) was reached after 12 hr.

**Figure 3:**
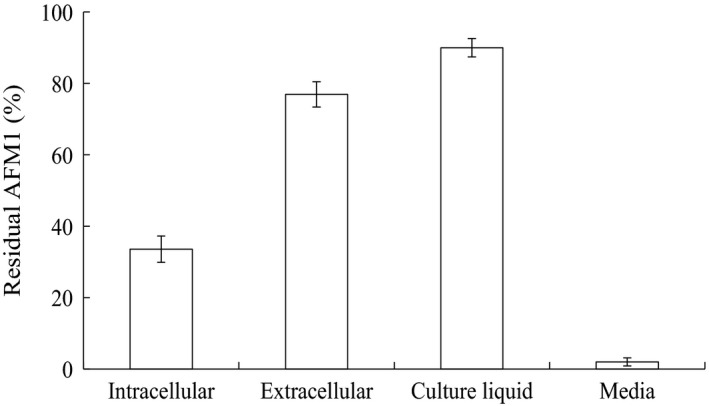
Aflatoxin M_1_ (AFM1) degradation by culture supernatant, cell and cell extracts of *Bacillus pumilus* E‐1‐1‐1

The culture supernatant of strain E‐1‐1‐1 could degrade 76.9% AFM_1_ compared to <5% and 10% by cell and cell extracts, respectively (Figure 3). These results showed that the detoxification mechanism of strain E‐1‐1‐1 was degradation rather than adsorption.

### 3.4 Effect of C source and N source on AFM_1_ degradation

**Figure 4:**
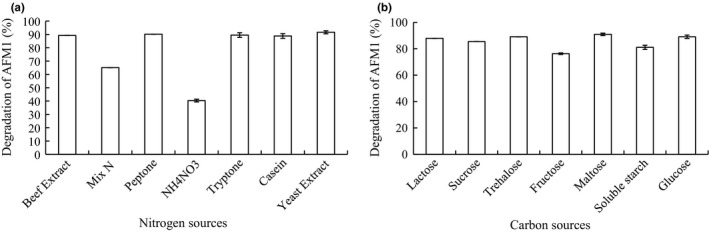
Effect of different carbon and nitrogen source on degradation of aflatoxin M_1_ (AFM1) (a) Effects of seven different carbon sources on the degradation of AFM1 by strain E‐1‐1‐1. (b) Effects of seven different nitrogen sources on the degradation of AFM1 by strain E‐1‐1‐1.

To determine the effect of C and N sources on AFM_1_ degradation, strain E‐1‐1‐1 was cultured in LBC medium or LBN medium. The resulting data are shown in Figure 4. Seven different N sources were tested. In general, higher AFM_1_ degradation ratios were observed in media with N sources of peptone, yeast extract, beef extract, tryptone, and casein. Biomass was also strongly influenced by N sources; yeast extract, beef extract, tryptone, and casein could significantly promote the biomass of strain E‐1‐1‐1. When strain E‐1‐1‐1 was cultured in media containing seven different C sources, the highest AFM_1_ degradation ratio was observed in media with the C source of maltose. Based on these results, maltose as C source and yeast extract as N source were selected as cultural conditions for production.

## 4 DISCUSSION

Aflatoxins appear in natural environments, but do not accumulate. This is because biological degradation occurs in nature. On this basis, microorganisms in animal feces, soils, and other environments can be chosen as sources for the selection of microorganisms that degrade aflatoxins (Chang & Lynd, 1970).

Due to short degradation time and nonpigmentation in foods, microbial degradation is preferred in the food and feed industry (Teniola et al., 2005). Scientific reports have shown that, to date, numerous microorganisms are capable of degrading aflatoxins (Adebo, Njobeh, Gbashi, Nwinyi, & Mavumengwanal, 2015). These bacterial species include *Nocardia corynebacteroides*,* Corynebacterium rubrum,* and *Rhodococcus* spp. (Ciegler, Lillehof, Peterson, & Hall, 1966). Of all the bacteria used to detoxify aflatoxins, lactic acid bacteria (LAB) are the most studied (Oliveira, Zannini, & Arendt, 2014). The ability of LABs to detoxify aflatoxins has been attributed to their strong affinity to the toxin (Hernandez‐Mendoza, Garcia, & Steele, 2009). Second, members of the genus *Bacillus* have been studied for their ability to detoxify aflatoxins. Farzaneh et al. (2012) reported that *Bacillus subtilis* UTBSP1 reduced aflatoxin B_1_ by 80.53% after incubation in the medium for 48 hr. We tested 68 isolates collected from animal waste, soil and activated sludge. The highest activity was detected in isolate E‐1‐1‐1, which was subsequently identified as *B. pumilus*. Strain E‐1‐1‐1 reduced AFM_1_ levels by 88.79% after incubation in the liquid medium at 37°C for 12 hr. Similar results were reported by other researchers (Smiley & Draughon, 2000; Teniola et al., 2005).

Our results demonstrated that C and N sources had highly significant effects on the degradation of AFM_1_ by *B. pumilus*. E‐1‐1‐1. In general, cultures with organic N sources gave higher detoxification than inorganic N sources.

The mechanism of biologically eliminating aflatoxins is by binding or degradation. Biological binding may be easily released and has a potential risk. Thus,the mechanism of strain E‐1‐1‐1 degrading toxins needs further study.

## 5 CONCLUSION

In this study, we obtained an AFM_1_‐degrading strain, *B. pumilus* E‐1‐1‐1, where the degradation ratio of AFM_1_ reached 89.55%. To the best of our knowledge, this is the first report that *B. pumilus* E‐1‐1‐1 possesses the ability to degrade AFM_1_. The detoxification of AFM_1_ by *B. pumilus* E‐1‐1‐1 is a rapid process, and AFM_1_ was decreased by 88.79% at 12 hr. Extracellular secretion from *B. pumilus* E‐1‐1‐1 caused an apparent decrease in AFM_1_ content. In conclusion, *B. pumilus* E1‐1‐1 could have great potential in the field of biological detoxification.

## CONFLICT OF INTEREST

The authors declare no conflict of interest.
